# Genetic variability, phylogeny and functional implication of the long control region in human papillomavirus type 16, 18 and 58 in Chengdu, China

**DOI:** 10.1186/s12985-020-01349-3

**Published:** 2020-07-16

**Authors:** Liyuan Fang, Xiaoli Lin, Yasi Yang, Zhilin Song, Xianping Ding, Liping Tan, Peng Gao

**Affiliations:** 1 Key Laboratory of Bio-Resource and Eco-Environment of Ministry of Education, College of Life Sciences, Sichuan University, Chengdu, 610065 Sichuan People’s Republic of China; 2 Bio-resource Research and Utilization Joint Key Laboratory of Sichuan and Chongqing, Chongqing, People’s Republic of China

**Keywords:** Human papillomavirus, Polymorphism, Phylogeny, Transcription factor binding sites prediction

## Abstract

**Background:**

Long control region (LCR) of human papillomavirus (HPV) has shown multiple functions on regulating viral transcription. The variations of LCR related to different lineages/sub-lineages have been found to affect viral persistence and cervical cancer progression differently. In this study, we focused on gene polymorphism of HPV16/18/58 LCR to assess the effect variations caused on transcription factor binding sites (TFBS) and provided more data for further study of LCR in Southwest China.

**Methods:**

LCR of HPV16/18/58 were amplified and sequenced to do polymorphic and phylogenetic anlysis. Sequences of each type were aligned with the reference sequence by MEGA 6.0 to identify SNPs. Neighbor-joining phylogenetic trees were constructed using MEGA 6.0. Transcription factor binding sites were predicted by JASPAR database.

**Results:**

The prevalence of these three HPVs ranked as HPV16 (12.8%) > HPV58 (12.6%) > HPV18 (3.5%) in Chengdu, Southwest China. 59 SNPs were identified in HPV16-LCR, 18 of them were novel mutations. 30 SNP were found in HPV18-LCR, 8 of them were novel. 55 SNPs were detected in HPV58-LCR, 18 of them were novel. Also, an insertion (CTTGTCAGTTTC) was detected in HPV58-LCR between position 7279 and 7280. As shown in the neighbor-joining phylogenetic trees, most isolates of HPV16/18/58 were clustered into lineage A. In addition, one isolate of HPV16 was classified into lineage C and 3 isolates of HPV58 were classified as lineage B. JASPAR results suggested that TFBS were potentially influenced by 7/6 mutations on LCR of HPV16/18. The insertion and 5 mutations were shown effects in LCR of HPV58.

**Conclusion:**

This study provides more data for understanding the relation among LCR mutations, lineages and carcinogenesis. It also helps performing further study to demonstrate biological function of LCR and find potential marker for diagnosis and therapy.

## Background

According to the data worldwide in 2012, cervical cancer is the fourth most common cancer in women, both for new cases and deaths. The data show large difference between developed and developing countries. It is the second most common cancer in less developed regions and covered 87% cervical cancer worldwide, and ranks 11th in developed regions [1]. There are an estimated 98,900 new cases and 30,500 deaths in China, 2015. That account for 18.7 and 11.5% of new cervical cancer cases and deaths worldwide, respectively [2].

Human papillomavirus (HPV) is a prevalent, globally distributed group of circular double-stranded DNA virus which can infect cutaneous and mucosal epithelia throughout the human body [3]. Persisting infection of high risk HPV is the most common reason to develop invasion cervical cancer that is confirmed by the majority. Over 220 HPV types have been fully characterized (https://www.hpvcenter.se/human_reference_clones/). Most oncogenic or high-risk HPV types are members of several species of Alpha-papillomavirus genus [4, 5] which are responsible for 90% of all cervical cancers worldwide [6]. Alpha-papillomavirus 7 is mostly related to high-risk mucosal lesion, including HPV18, HPV45, HPV39, etc. Alpha-papillomavirus 9 is the most important species to malignant mucosal lesions, including HPV16, HPV33, HPV58, etc. [7]. HPV16/18 are associated with approximately 70% of invasion cervical cancers worldwide, making them the primary targets for research and vaccination alike [8]. Compelling data demonstrate that HPV16 associated with persistence of infection, development of precancer, progression and histologic type of cervical cancer [9]. The relationship between HPV18 and precancerous lesion is not as compelling as HPV16, but many study have suggested the relevance between HPV18 and cancer [10, 11]. HPV58 has a especially high prevalence in East Asia and ranks the third in cervical cancer cases [12]. Furthermore, it is also frequently appears in precancerous lesions, even more than HPV18, and takes the second slot (overall in 21.1% of CIN2/3) [13].

The whole genome of HPV contains three regions, an early region (E1, E2, E3, E4, E5, E6, E7), a late region (L1, L2) and a regulatory region called long control region or upstream regulatory region (LCR or URR) [14]. The LCR is an around 850 bp non-coding sequence which has active interaction with many cellular and viral factors. This region include the viral early promoter and transcriptional enhancer, the viral origin of replication, the late polyadenylation site and the late (or negative) regulatory element (LRE/NRE). In this way, it can control late gene expression at various post-transcriptional levels [15].

The LCR has been shown to be the most variable region of HPV genome, mainly because it does not encode any gene and therefore able to accumulate and tolerate more mutations [16, 17]. The mutations in this region divide HPV into different lineages and sub-lineages which perform differently in viral persistence and progression of precancer/cancer. In HPV16, non-European (sub-lineage A4, B, C, D) variants get three-fold or higher risk to associated with cervical cancer than European (Sub-lineage A1-A3) variants. Non-European variants of HPV18 are also detected more commonly in cancer tissues and high grade cervical lesions [18–20]. HPV58 is the second common HPV-type in Southwest China in previous data [21, 22], the variants (C632T and G760A, located on E7) of which have been reported to be highly associated with cervical cancer [23]. The LCR variants have been shown to differently regulate the replication of HPV throughout the viral life cycle [13] and the transcriptional activity of E6 and E7 [14].

In this study, we collected the positive samples of HPV16, 18, 58. Analysis of polymorphism, phylogenetic and functional prediction were performed on LCR which were rarely reported in Chengdu, Southwest China. The data helped us to determine the prevalence of lineages/sub-lineages and novel mutations/isolates of each type. It is useful for epidemiological survey and biological function research of LCR.

## Methods

### Ethical approval and consent to participants

This study was approved by education and research committee and Ethics Committee of Sichuan University, China (approval number SCU20100196494). All works were followed the guideline of Ethics Committee of Sichuan University. Informed Consent Right was confirmed by patients enrolling. The privacy of patients was assured to protect carefully.

### Samples

All 8244 gynecological outpatients’ cervical swab samples were collected between September 2017 and June 2019 in Chengdu SongZiNiao Sterility Hospital, Sichuan Reproductive Health Research Center Affiliated Hospital, Chengdu Western Hospital Maternity Unit, and Angel Women’s and Children’s Hospital. The samples were collected from women aged 20 to 59 who have normal cytology, low-grade squamous intraepithelial lesion or cervical intraepithelial neoplasia. Each sample was stored at − 20 °C in cell preservation fluid. Specimen collection to DNA extraction was in a week.

### DNA extraction and HPV typing

DNA was extracted using nucleic acid extraction kit (Health gene technologies, Ningbo, China) in accordance with the manufacturer’s instructions. Via capillary electrophoresis method by multiple PCR, DNA extraction was amplified using HPV nucleic acid assay and genotyping kit (Health gene technologies, Ningbo, China). Genotyping was accomplished by Applied Biosystems 3500Dx genetic analyzer. The kit can distinguish 25 types (HPV6, 11, 16, 18, 26, 31, 33, 35, 39, 42, 43, 44, 45, 52, 53, 56, 58, 59, 66, 68, 73, 81, 82, 83) of HPV. HPV 16/18/58 positive samples were selected for the following study.

### PCR amplification of HPV-LCR

LCR sequences of HPV16/18/58 were amplified by primers which were designed based on the reference sequences from GenBank (Table 1). PCRs were performed in a final volume of 30 μL, containing 2*PCR buffer (200 mM Tris–HCl pH 8.3; 200 mM KCl), 2.5 mM dNTPs, 2 U of EasyTaq DNA Polymerase, and 0.5 μM of each primer of the pairs. The PCR program was set as following conditions: an initial denaturation at 95 °C for 5 min, then entered 30 amplification cycles, 95 °C for 30 s, primer annealing at 45 ~ 55 °C (52 °C for HPV18 and HPV58, 45 °C for HPV16) for 30s and elongation at 72 °C for 30 s, after 7 min final extension at 72 °C, ended up and held the temperature at 4 °C. The PCR products were detected using ChemiDoc XRS+ imaging system (Bio-Rad Laboratory, Mississauga, Canada) after electrophoresis through 1.5% agarose gel. The positive DNA fragments were purified and sequenced by TSINGKE, China.

**Table 1:** Primer pairs designed for HPV 16/18/58 LCR amplification

Primer name	Primer sequence	Product size	Anneal
HPV-16 R	5′ CACCACCTCATCTACCTCT 3’	909 bp	48 °C
HPV-16 F	5′ ACATTGCAGTTCTCTTTTG 3’		
HPV-18 R	5′ TTTTGGTTCAGGCTGGATT 3’	989 bp	54 °C
HPV-18 F	5′ AGGTAGCTTGTAGGGCTGC 3’		
HPV-58 R	5′ CATCCACCAAACGCAAA 3’	883 bp	53 °C
HPV-58 F	5′ TCCAACGCCTGACACAA 3’		

### Analysis of DNA sequences

To identify the single nucleotide polymorphisms (SNP) in LCR, HPV prototype reference sequences were used as the standard to compare with the valid LCR sequences of each type by MEGA 6.0 [24]. The phylogenetic trees were constructed by the Neighbor-Joining method using Kimura 2-Parameter model. The number of bootstrap replications was set at 1000. Sub-lineage reference sequences of the specific type of HPV were participated in constructing the branches of phylogenetic tree (Table 2). All sequences were analyzed using the BLAST (Basic Local Alignment Search Tool) from NCBI (https://blast.ncbi.nlm.nih.gov/Blast.cgi) to detect the novel sites or isolates.

**Table 2:** Reference sequences of LCR

HPV type	Sub-lineages Genbank accession No.									
**HPV16**	**A1**	**A2**	**A3**	**A4**	**B1**	**B2**	**C**	**D1**	**D2**	**D3**
	K027189	AF536179	HQ644236	AF534061	AF536180	HQ644298	AF472509	HQ644257	HQ644270	AF402678
**HPV18**	**A1**	**A2**	**A3**	**A4**	**A5**	**B1**	**B2**	**B3**	**C**	
	AY262282	EF202146	EF202147	EF202151	GQ180787	EF202155	KC470225	EF202152	KC470229	
**HPV58**	**A1**	**A2**	**A3**	**B1**	**B2**	**C**	**D1**	**D2**		
	D90400	HQ537752	HQ537758	HQ537762	HQ537764	HQ537774	HQ537768	HQ537770		

### Prediction of transcription factor binding sites

The online database JASPAR (http://jaspar.genereg.net/) were used to predicted transcription factor binding sites (TFBS) in LCR of HPV16/18/58 [25]. Transcription factors that we were interested in were as follows: CEBPB, ETS1, FOS, FOXA1, HSF1, HOXC11,IRF1, IRF2, IRF7, JUN, MAFK, NFIL3, NFKB1, PHOX2A, RAX, SPIB, STAT1, STAT3, SRF, SP1, SRY, SOX9, VAX1 and YY1 for HPV16; CEBPB, ETS1, FOXA1, FOXC1, FOS, GATA3, HOXA10, HOXC11, HOXB13, ID4, JUN, KLF5, NKX6–1, RUNX1, RHOXF1, RAX, SRF, SRY, SOX10, SNAI2, TBR1, TEAD1, VAX1 and YY1 for HPV18; CEBPA, CREB1, ESR2, ETS1, ELK4, FOS, FOXP3, HSF1, HOXC11, IRF2, JUN, NFKB1, NFIA, PHOX2A, POU2F2, RAX, SPIB, SMAD3, STAT1, SOX9, SOX10, SRY, VAX1 and YY1,for HPV58. The relative profile score threshold was set at 85%.

## Results

### Characteristics of HPV prevalence in Southwest China

**Fig. 1:**
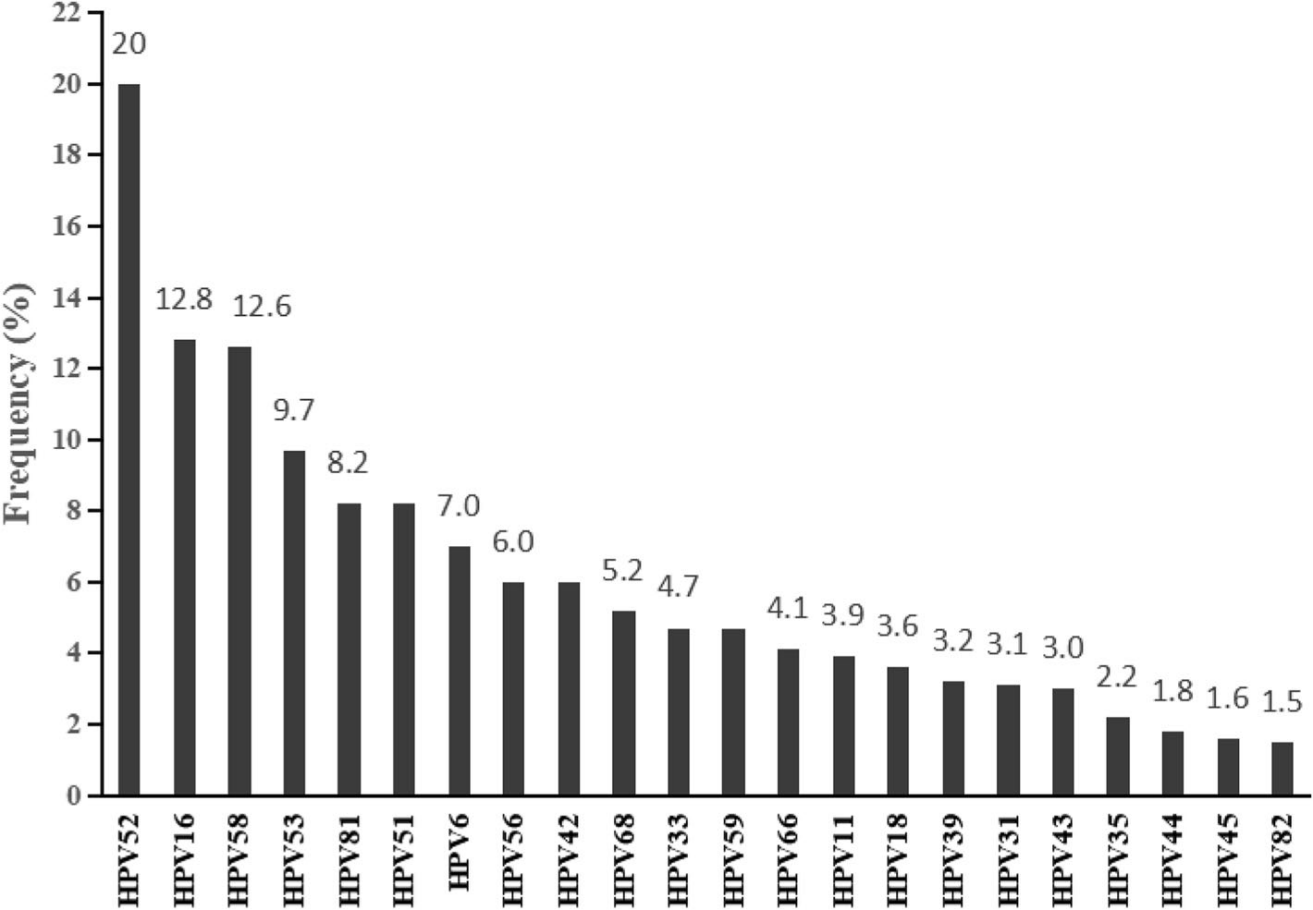
Prevalence of HPV in Chengdu, Sichuan

Total 8244 samples were collected from September 2017 to June 2019, 2367 (28.7%) of them were HPV-positive. Among them, 302(12.8%), 82(3.5%) and 299(12.6%) were tested as HPV16, HPV18 and HPV58, respectively (Fig. 1). The identical sequence was regarded as a pattern. In HPV18, 16 patterns were confirmed from 34 sequences. Pattern No.2 (12 isolates, 35.3%) was the most prevalence pattern (Table 3). In 57 HPV16 LCR sequences, pattern No.6 (6 isolates, 10.2%) was the most common pattern from the 22 patterns (Table 4). Total 31 patterns were identified in 66 HPV58 samples. Pattern No.5 (16 isolates, 24.2%) was completely same with HPV58 reference sequence which was also the most common pattern (Table 5).

**Table 3:** HPV18 LCR nucleotide variant sites and pattern number

Nucleotide positon	7140	7164	7184	7258	7403	7486	7524	7529	7530	7563	7564	7567	7592	7670	7694	7716	7720	7753	7754	7853	7857	2	19	24	33	41	53	55	69	104	Pattern	
	G	C	C	T	A	C	G	C	T	G	A	A	T	A	T	C	C	T	C	T	C	T	T	T	A	A	T	G	T	T	No.	amount
**HPV18 NC01**	–	–	–	–	–	–	–	–	–	–	–	–	C	–	–	–	–	–	–	G	–	–	–	–	–	–	–	–	–	–	1	2
**HPV18 NC02**	–	–	–	–	–	–	–	–	–	–	–	–	C	–	–	–	–	–	–	–	–	–	–	–	–	–	–	–	–	–	2	12
**HPV18 NC03**	–	–	–	A	–	T	–	A	–	–	–	C	C	T	–	–	–	–	–	–	–	–	–	–	–	–	–	–	–	C	3	3
**HPV18 NC05**	–	–	–	–	–	–	A	–	–	–	–	–	C	–	–	–	–	–	–	–	–	–	–	–	–	–	–	–	–	–	4	2
**HPV18 NC06**	–	–	–	A	–	–	–	A	–	–	–	C	C	T	–	–	–	G	T	–	–	–	–	–	–	G	–	–	–	C	5	1
**HPV18 NC07**	–	–	–	–	–	–	–	–	–	–	–	–	C	–	–	–	–	–	–	–	–	–	G	–	–	–	–	–	–	–	6	2
**HPV18 NC11**	–	–	–	–	C	–	–	–	–	–	–	–	C	–	–	–	–	–	–	–	–	–	–	–	–	–	–	–	–	–	7	2
**HPV18 NC14**	A	–	–	–	–	–	–	–	–	–	–	–	C	–	–	–	–	–	–	–	–	–	–	–	–	–	–	–	–	–	8	2
**HPV18 NC15**	–	–	–	–	–	–	–	–	–	–	–	–	C	–	–	–	–	–	–	–	–	–	–	–	T	–	–	–	–	–	9	1
**HPV18 NC16**	–	–	–	–	–	–	–	–	–	–	–	–	C	–	–	–	–	–	–	–	T	–	–	–	–	–	G	C	G	–	10	1
**HPV18 NC19**	–	–	–	–	–	–	–	–	–	–	–	–	C	–	–	–	–	–	–	–	–	G	–	–	–	–	–	–	G	–	11	1
**HPV18 NC20**	–	–	–	–	–	–	–	–	–	–	–	–	C	–	–	–	–	–	–	–	–	–	–	A	–	–	–	–	–	–	12	1
**HPV18 NC21**	–	–	–	–	–	–	–	–	–	–	–	–	C	–	–	–	–	–	–	–	–	–	–	–	–	–	–	–	G	–	13	1
**HPV18 NC25**	–	–	–	–	–	–	–	–	–	–	–	–	C	–	–	–	–	–	–	–	T	–	–	–	–	–	–	–	–	–	14	1
**HPV18 NC33**	–	–	–	A	–	–	–	A	A	A	C	C	C	T	G	T	A	–	–	–	–	–	–	–	–	–	–	–	–	–	15	1
**HPV18 NC34**	–	A	T	A	–	T	–	A	–	A	–	C	C	T	–	–	–	–	–	–	–	–	–	–	–	–	–	–	–	–	16	1

Same nucleotide compared to the reference sequence were marked with a dash (−). N noted novel variants of this study. We gave a specific pattern No. to identical LCR sequence. The sample amounts of each pattern were listed at last column

**Table 4:** HPV16 LCR nucleotide variant sites and pattern number

Nucleotide Positon	7168	7174	7175	7177	7178	7193	7201	7210	7219	7233	7238	7252	7270	7287	7289	7292	7310	7328	7343	7366	7385	7394	7395	7419	7428	7429	7435	7449	7458	7483	7485	7489	7495	7521	7595	7660	7669	7714	7730	7765	7770	7775	7777	7781	7785	7816	7826	7830	7834	7837	7839	7842	7864	7868	7873	7874	Pattern	
	A	A	A	T	A	G	T	C	G	A	T	T	C	A	A	C	C	T	C	C	T	C	C	A	C	G	G	T	A	A	A	G	T	G	C	A	C	T	A	T	C	T	A	T	C	G	G	A	G	A	A	G	T	G	A	C	No	A
**HPV16 NC01**	-	-	C	C	-	T	C	-	-	-	-	-	T	C	-	-	-	-	-	-	-	-	-	-	-	-	-	-	-	-	-	-	-	A	-	G	-	-	C	-	T	-	-	-	-	C	-	C	-	C	-	A	C	-	-	-	1	2
**HPV16 NC02**	-	-	C	C	-	T	C	-	-	-	-	-	T	C	C	-	-	-	-	-	-	-	-	-	-	A	-	-	-	-	-	-	-	A	-	-	-	-	C	-	-	-	-	-	-	-	-	-	-	-	-	A	-	-	-	T	2	1
**HPV16 NC03**	-	-	C	C	-	T	C	-	-	-	-	-	T	C	-	-	-	-	-	-	-	-	-	-	-	-	-	-	-	-	-	-	-	A	-	-	-	-	C	C	-	C	C	-	-	C	-	-	-	C	C	A	-	-	-	-	3	2
**HPV16 NC04**	-	-	C	C	-	T	C	-	-	-	-	-	T	C	C	-	-	-	--	-	-	-	-	-	-	A	-	-	-	-	-	-	-	A	-	-	-	-	C	-	-	-	-	C	-	-	-	-	-	-	-	A	-	-	-	-	4	1
**HPV16 NC06**	-	-	C	C	-	T	C	-	-	-	-	-	T	C	C	-	-	-	-	-	-	-	-	-	-	-	-	-	-	-	-	-	-	A	-	-	-	-	C	-	-	-	-	-	-	-	-	C	-	-	-	A	-	A	-	-	5	1
**HPV16 NC07**	-	-	C	C	-	T	C	-	-	-	-	-	T	C	C	-	-	-	-	-	-	-	-	G	-	-	-	-	-	-	-	-	-	A	-	-	-	-	C	-	-	-	-	-	-	-	-	-	-	-	-	A	-	-	-	-	6	6
**HPV16 NC08**	-	-	C	C	-	T	C	-	-	-	-	-	T	C	C	-	-	-	-	-	-	-	-	-	-	-	-	-	-	-	-	-	-	A	-	-	-	-	C	-	-	-	-	C	-	-	-	-	-	-	-	A	-	-	-	-	7	3
**HPV16 NC09**	-	-	C	C	-	T	C	A	-	G	-	-	T	C	C	-	-	-	-	-	-	-	-	-	-	-	-	-	-	-	-	-	-	A	-	-	-	-	C	-	-	-	-	C	-	-	-	-	-	-	-	A	-	-	-	-	8	1
**HPV16 NC10**	-	-	C	C	-	T	C	-	-	-	-	-	T	C	-	-	-	-	-	-	-	-	-	-	-	-	-	-	-	-	-	-	-	A	-	G	-	-	C	-	-	-	-	-	-	-	-	-	-	-	-	A	-	-	-	-	9	3
**HPV16 NC11**	-	-	C	C	-	T	C	A	-	-	-	-	T	C	C	-	-	-	-	-	-	-	-	-	-	-	-	-	-	-	-	-	-	A	-	-	-	-	C	-	-	-	-	-	-	-	-	-	-	-	-	A	-	-	-	-	10	1
**HPV16 NC12**	-	-	C	C	-	T	C	-	-	-	-	-	T	C	C	-	-	-	-	-	-	-	-	-	-	A	-	-	-	-	-	-	-	A	-	-	-	-	C	-	-	-	-	-	-	-	-	-	-	-	-	A	-	-	-	T	11	1
**HPV16 NC13**	-	-	C	C	-	T	C	-	-	-	-	-	T	C	-	-	-	-	-	-	-	-	-	-	-	-	-	-	-	-	-	-	-	A	-	-	-	-	C	-	-	-	-	-	-	-	-	-	-	-	-	A	-	-	G	-	12	1
**HPV16 NC15**	-	-	C	C	-	T	C	-	-	-	-	-	T	C	-	-	-	-	-	-	-	-	-	-	-	-	-	-	-	-	-	-	-	A	-	-	-	-	C	-	-	-	-	-	-	-	-	-	-	-	-	A	-	-	-	-	13	3
**HPV16 NC17**	-	-	C	C	-	T	C	-	C	-	-	-	T	T	C	-	-	-	-	-	-	-	-	-	A	-	-	-	-	-	-	-	-	A	-	-	-	-	C	-	-	-	-	C	-	-	-	-	-	-	-	A	-	-	-	-	14	1
**HPV16 NC19**	-	-	C	C	-	T	C	-	-	-	-	-	T	T	-	-	-	-	-	G	-	A	-	-	-	-	-	-	-	-	-	-	-	A	T	G	-	-	C	-	-	-	-	-	-	-	-	-	-	-	-	A	-	-	-	-	15	1
**HPV16 NC24**	-	-	C	C	-	T	C	-	-	-	-	-	T	C	-	-	-	-	-	-	G	-	-	-	-	-	-	-	-	-	-	-	-	A	-	-	-	-	C	-	-	-	-	-	-	-	-	-	-	-	-	A	-	-	G	-	16	1
**HPV16 NC25**	-	-	C	C	-	T	C	-	-	-	-	-	T	C	C	-	-	-	-	-	-	-	-	-	-	A	-	-	-	-	-	-	-	A	-	-	-	-	C	-	-	-	-	-	-	-	-	-	-	-	-	A	-	-	-	G	17	3
**HPV16 NC30**	-	-	-	-	-	T	-	-	-	-	-	-	-	-	-	-	-	-	-	-	-	-	-	-	-	-	-	-	-	-	-	-	-	A	-	-	-	G	-	-	-	-	-	-	-	-	-	-	-	-	-	-	-	-	-	-	18	5
**HPV16 NC32**	G	C	-	-	-	T	-	-	-	-	-	-	-	-	-	-	-	-	-	-	-	-	-	-	-	-	-	-	-	-	-	-	-	A	-	-	-	-	-	-	-	-	-	-	-	-	-	-	-	-	-	-	-	-	-	-	19	2
**HPV16 NC33**	G	-	-	-	-	T	-	-	-	-	-	-	-	-	-	-	-	-	-	-	-	-	-	-	-	-	-	-	-	-	-	-	-	A	-	-	-	-	-	-	-	-	-	-	-	-	-	-	-	-	-	-	-	-	-	-	20	1
**HPV16 NC35**	G	-	-	-	-	T	-	-	-	-	-	-	-	-	-	-	T	-	-	-	-	-	T	-	-	-	-	-	-	C	-	-	-	A	-	-	-	-	-	-	-	-	-	-	-	-	-	-	-	-	-	-	-	-	-	-	21	1
**HPV16 NC36**	G	-	-	-	-	T	-	-	-	-	-	-	-	-	-	-	T	-	-	-	-	-	T	-	-	-	-	-	-	-	-	-	-	A	-	-	-	-	-	-	-	-	-	-	-	-	-	-	-	-	-	-	-	-	-	-	22	3
**HPV16 NC37**	-	-	-	-	G	T	-	-	-	-	-	-	-	-	-	-	-	-	T	-	-	-	-	-	-	-	-	-	-	-	-	-	-	A	-	-	-	C	-	-	-	-	-	-	-	C	-	-	-	-	-	-	-	-	-	-	23	2
**HPV16 NC38**	-	-	-	-	-	T	-	-	-	C	-	-	-	-	-	-	-	-	-	-	-	-	-	-	-	-	-	-	-	-	-	-	-	A	-	-	-	G	-	-	-	-	-	-	-	-	-	-	-	-	-	-	-	-	-	-	24	1
**HPV16 NC40**	-	-	-	-	-	T	-	-	-	-	A	-	-	-	-	-	-	-	-	-	-	-	-	-	-	-	-	-	-	-	-	-	C	A	-	-	-	-	-	-	-	-	-	-	-	-	-	-	-	-	-	-	-	-	-	-	25	1
**HPV16 NC41**	-	-	-	-	-	T	-	-	-	-	-	-	-	-	-	-	-	-	-	-	-	-	-	-	-	-	-	G	-	-	-	-	-	A	-	-	-	-	-	-	-	-	-	-	-	-	-	-	-	-	-	-	-	-	-	-	26	1
**HPV16 NC42**	-	-	-	-	-	T	-	-	-	-	-	-	-	-	-	A	-	-	-	-	-	-	-	-	-	-	-	-	-	-	-	-	-	A	-	-	-	-	-	-	-	-	-	-	-	-	-	-	-	-	-	-	-	-	-	-	27	1
**HPV16 NC43**	-	-	-	-	-	T	-	-	-	C	-	-	-	-	-	-	-	-	-	-	-	-	-	-	-	-	A	-	T	-	C	A	-	A	-	-	T	-	C	-	-	-	-	-	T	-	A	-	T	C	G	A	-	-	-	-	28	1
**HPV16 NC44**	-	-	-	-	-	T	-	-	-	-	-	-	-	-	-	-	-	C	-	-	-	-	-	-	-	-	-	-	-	-	-	-	-	A	-	-	-	-	-	-	-	-	-	-	-	-	-	-	-	-	-	-	-	-	-	-	29	1
**HPV16 NC45**	-	-	-	-	-	-	-	-	-	-	-	-	-	-	-	-	-	-	-	-	-	-	-	-	-	-	-	-	-	-	-	-	-	-	-	-	-	-	-	-	-	-	-	-	-	-	-	-	-	-	-	-	-	-	-	-	30	1
**HPV16 NC46**	-	-	-	-	-	T	-	-	-	-	-	-	-	-	-	-	-	-	-	-	-	-	-	-	-	-	-	-	-	-	-	-	-	A	-	-	-	-	-	-	-	-	-	-	-	-	-	-	-	-	-	-	-	-	-	-	31	3
**HPV16 NC47**	-	-	-	-	-	T	-	-	-	-	-	C	-	-	-	-	-	-	-	-	-	-	-	-	-	-	-	-	-	-	-	-	-	A	-	-	-	-	-	-	-	-	-	-	-	-	-	-	-	-	-	-	-	A	-	-	32	1

Same nucleotides compared to the reference sequence were marked with a dash (-). N noted novel variants of this study. We gave a specific pattern No. to identical LCR sequence. The sample amounts of each pattern were listed at last column

**Table 5:** HPV58 LCR nucleotide variant sites and pattern number

Nucleotide Positon	7147	7148	7154	7175	7186	7188	7194	7199	7207	7211	7257	7265	7266	7279	7280	7284	7304	7313	7376	7421	7431	7435	7464	7522	7525	7540	7619	7621	7673	7714	7730	7745	7749	7755	7779	7791	7793	18	20	30	31	33	36	52	53	54	62	65	86	88	92	97	102	Pattern	
	G	G	A	T	A	G	G	C	T	G	T	C	C	-		C	A	A	A	G	T	A	T	T	A	A	G	A	A	A	C	G	C	A	A	T	A	A	C	C	T	G	T	C	T	G	C	G	T	T	G	C	G	No.	amount
**HPV58 NC01**	-	-	-	-	-	-	C	-	-	-	-	-	-	+		-	G	-	-	-	-	-	-	-	C	-	-	-	-	C	-	-	-	G	-	-	C	-	-	-	C	-	-	T	-	-	-	-	-	-	-	-	-	1	1
**HPV58 NC02**	-	-	-	-	-	-	-	-	-	-	-	-	T	-		-	-	-	-	-	-	-	-	-	-	-	-	-	C	-	-	-	-	-	-	-	-	-	-	-	-	-	-	-	A	-	-	-	-	-	-	-	-	2	1
**HPV58 NC03**	-	-	-	-	-	-	-	-	-	-	-	-	-	-		-	-	-	-	-	-	-	-	-	-	-	-	-	-	-	-	-	-	-	-	-	-	-	-	-	-	-	-	-	-	-	-	-	-	-	-	-	-	3	16
**HPV58 NC04**	-	-	-	-	-	-	-	-	-	-	-	G	T	-		-	-	-	-	-	-	-	-	-	-	-	-	-	-	G	-	-	-	-	-	-	-	-	-	-	-	-	-	T	-	-	-	-	-	-	-	-	-	4	3
**HPV58 NC07**	-	-	-	-	-	-	-	-	-	-	-	-	-	-		-	-	-	-	-	-	-	-	-	-	-	-	-	-	-	-	-	-	-	-	-	-	-	-	A	-	-	-	-	-	-	-	-	-	-	-	-	-	5	1
**HPV58 NC08**	-	-	-	-	-	-	-	-	-	-	-	-	-	-		-	-	-	-	-	-	-	-	-	-	-	-	-	-	-	-	-	-	-	-	-	G	-	-	-	-	-	-	-	-	-	-	-	-	-	-	-	-	6	6
**HPV58 NC10**	-	-	-	-	-	-	-	-	-	-	-	-	-	-		-	-	-	-	-	-	-	-	-	-	-	-	-	-	T	-	-	-	-	-	-	-	-	-	-	-	-	-	-	-	-	-	-	-	-	-	-	-	7	2
**HPV58 NC11**	-	-	-	-	-	-	-	-	-	-	-	-	-	-		-	-	-	-	-	-	-	-	-	-	G	-	-	-	-	-	-	G	-	-	-	-	-	-	-	-	-	-	-	-	-	-	-	-	-	-	-	-	8	4
**HPV58 NC12**	-	-	-	-	-	-	-	-	-	-	-	-	T	-		-	-	-	-	-	-	-	-	-	-	-	-	-	-	G	-	-	-	-	-	-	-	-	-	-	-	-	-	-	-	-	-	-	-	-	-	-	-	9	1
**HPV58 NC16**	-	-	-	-	C	-	-	T	A	-	-	-	-	-		G	-	-	-	-	-	G	-	C	-	-	-	-	-	-	A	C	-	-	G	-	-	-	-	G	-	-	-	T	-	A	A	-	-	-	-	T	-	10	1
**HPV58 NC17**	-	-	-	-	-	-	-	-	-	-	-	G	T	-		-	-	-	-	-	-	-	-	-	-	-	-	-	-	G	-	-	-	-	-	-	-	-	-	-	-	-	-	-	-	-	-	-	-	-	-	-	-	11	5
**HPV58 NC20**	-	-	-	-	-	-	-	-	-	T	-	-	-	-		-	-	-	-	-	-	-	-	-	-	-	-	T	-	-	-	-	-	-	-	-	-	-	-	-	-	-	-	-	-	-	-	-	-	-	-	-	-	12	1
**HPV58 NC23**	-	-	-	-	C	-	-	T	A	-	G	-	-	-		-	-	C	-	-	-	-	-	C	-	G	A	-	-	-	-	-	-	-	-	-	-	-	-	G	-	-	-	T	-	A	-	-	-	-	-	-	-	13	2
**HPV58 NC26**	-	-	-	-	-	A	-	-	-	-	-	G	T	-		-	-	-	-	-	-	-	-	-	-	-	-	-	-	G	-	-	-	-	-	-	-	-	-	-	-	-	-	T	-	-	-	-	-	-	-	-	-	14	3
**HPV58 NC29**	-	-	-	-	-	-	C	-	-	-	-	-	-	+		-	G	-	-	-	-	-	-	-	-	-	-	-	-	C	-	-	-	G	-	-	-	-	-	-	-	-	-	T	-	-	-	-	-	-	-	-	-	15	3
**HPV58 NC33**	T	-	-	-	-	-	C	-	-	-	-	-	-	+		-	G	-	-	-	-	-	-	-	-	-	-	-	-	C	-	-	-	G	-	-	-	-	-	-	-	-	-	T	-	A	-	-	G	-	-	-	-	16	1
**HPV58 NC34**	T	-	-	-	-	-	C	-	-	-	-	-	-	+		-	G	-	-	-	-	-	-	-	-	-	-	-	-	C	-	-	-	G	-	-	-	-	-	-	-	-	-	T	-	-	-	-	-	-	-	-	-	17	1
**HPV58 NC35**	T	-	-	-	-	-	C	-	-	-	-	-	-	+		-	G	-	-	-	-	-	-	-	C	-	-	-	-	C	-	-	-	G	-	-	C	-	-	-	-	-	-	T	-	-	-	-	-	-	-	-	-	18	1
**HPV58 NC37**	T	-	-	-	-	-	C	-	-	-	-	-	-	+		-	G	-	-	-	-	-	-	-	C	-	-	-	-	C	-	-	-	G	-	-	C	C	-	-	-	-	-	T	-	-	-	-	-	-	-	-	-	19	1
**HPV58 NC41**	-	-	-	-	-	-	-	-	-	-	-	-	-	-		-	-	-	-	C	-	-	-	-	-	-	-	-	-	-	-	-	-	-	-	-	G	-	G	-	-	-	-	-	-	-	-	-	-	-	-	-	-	20	1
**HPV58 NC46**	-	C	-	-	-	-	-	-	-	-	-	G	T	-		-	-	-	-	-	-	-	-	G	-	-	-	-	-	G	-	-	-	-	-	-	-	-	-	-	-	-	-	-	-	-	-	-	-	-	-	-	-	21	1
**HPV58 NC49**	-	-	-	-	-	-	-	-	-	-	-	-	-	-		-	-	-	G	-	-	-	G	-	-	-	-	-	-	-	-	-	-	-	-	-	-	-	-	-	-	-	-	-	-	-	-	-	-	-	-	-	-	22	1
**HPV58 NC50**	-	-	-	-	-	-	-	-	-	-	-	-	-	-		-	-	-	-	-	-	-	-	-	-	G	-	-	-	-	-	-	G	-	-	-	-	-	-	-	-	-	-	T	-	-	-	-	-	-	-	-	-	23	1
**HPV58 NC52**	-	-	-	-	-	-	-	-	-	-	-	-	-	-		-	-	-	-	-	-	-	-	-	-	-	-	-	-	-	-	-	-	-	-	A	C	-	-	-	-	T	C	-	-	-	-	C	-	-	T	-	-	24	1
**HPV58 NC53**	-	-	-	-	-	-	-	-	-	-	-	-	-	-		-	-	-	-	-	-	-	-	-	-	G	-	-	-	-	-	-	-	-	-	-	-	-	-	-	-	-	-	-	-	-	-	-	-	-	-	-	-	25	1
**HPV58 NC56**	-	-	-	C	-	-	-	-	-	-	-	-	-	-		-	-	-	-	-	-	-	-	-	-	-	-	-	-	-	-	-	-	-	-	-	-	-	-	-	-	-	-	T	-	-	-	-	-	-	-	-	-	26	1
**HPV58 NC57**	-	C	-	-	-	-	-	-	-	-	-	-	-	-		-	-	-	-	-	-	-	-	-	-	-	-	-	-	-	-	-	-	-	-	-	G	-	-	-	-	-	-	-	-	-	-	-	-	-	-	-	-	27	1
**HPV58 NC59**	-	-	-	-	-	-	-	-	-	-	-	-	-	-		-	-	-	-	-	-	-	-	-	-	-	-	-	-	-	-	-	-	-	-	-	G	-	-	-	-	-	-	-	-	-	-	-	-	-	-	-	A	28	1
**HPV58 NC61**	-	-	-	-	-	-	-	-	-	-	-	-	T	-		-	-	-	-	-	G	-	-	-	-	-	-	-	-	G	-	-	-	-	-	-	-	-	-	-	-	-	-	-	-	-	-	-	-	-	-	-	-	29	1
**HPV58 NC63**	-	-	-	-	-	-	-	-	-	-	-	-	T	-		-	-	-	-	-	-	-	-	-	-	-	-	-	-	G	-	-	-	-	-	-	-	-	-	-	-	-	-	-	-	-	-	-	-	C	-	-	-	30	1
**HPV58 NC65**	-	-	T	-	-	-	-	-	-	-	-	-	-	-		-	-	-	-	-	-	-	-	-	-	G	-	-	-	-	-	-	G	-	-	-	-	-	-	-	-	-	-	-	-	-	-	-	-	-	-	-	-	31	1

Same nucleotides compared to the reference sequence were marked with a dash (-). N noted novel variants of this study. + present the insert (CTTGTCAGTTTC) between 7279 and7280. We gave a specific pattern No. to identical LCR sequence. The sample amounts of each pattern were listed at last column

### Genomic polymorphisms of HPV-LCR

30 SNPs were identified in HPV18-LCR. T7592C was found in all isolates. T7258A, C7529A, A7567C and A7670T was the second common mutations in HPV18-LCR, got the frequency of 17.6%. These four mutations also appeared together. No insertion or deletion was found. After Blast on NCBI, 8 unique mutations and 9 novel variants were confirmed. (Table 3).

59 SNPs were detected in 56 nucleotide positions of HPV16 LCR. The most common mutations, G7193T and G7521A was found in all isolates except HPV16 NC45. A7730C and G7842A were identified in 57.9% of the variants (33/57). A7175C, T7177C, T7201C and C7270T were detected in 32 isolates. No insertion or deletion mutation was found. After Blast on NCBI, we found that 18 mutations and 9 variants were never reported by anyone else. (Table 4).

In HPV58, 55 SNPS were found at 52 nucleotide positions. An insertion (CTTGTCAGTTTC) was detected between the nucleotide sites 7279 and 7280. The most variable site was 7714 (25/67). All four kinds of nucleotide were found on this position, including 8 of A7714C, 15 of A7714G and 2 of A7714T. The second most prevalence mutation was C52T (19/67). Two groups of mutations had a tendency to show together, which were C7265G (12/67), A7714G (15/67) and C7266T (16/67); G7194C (8/57), A7304G (8/57), the insertion (8/57) and A7714C (8/67). 18 novel sites were found in HPV58 which composed 12 novel sequences. (Table 5).

### Phylogenetic analysis

The neighbor-joining phylogenetic trees were constructed by MEGA, using the patterns and reference sequences of sub-lineages .

**Fig. 2:**
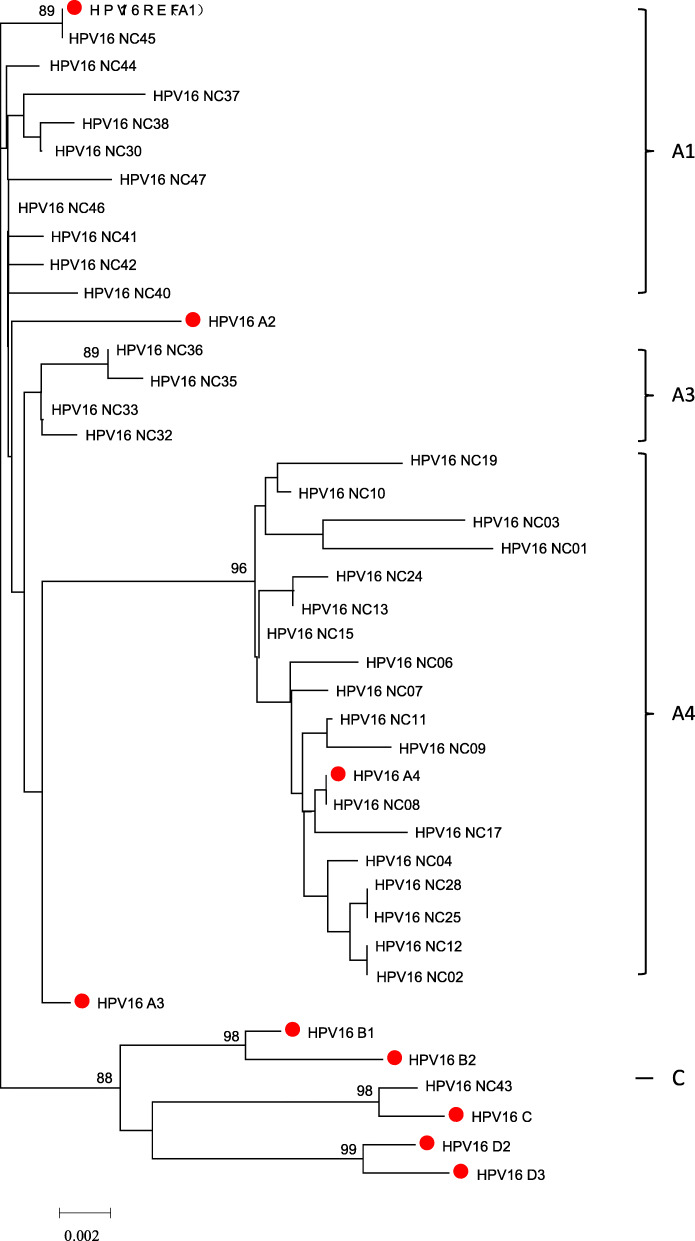
Neighbor-joining phylogenetic tree of HPV-16 patterns based on LCR sequences. Reference sequences of sub-lineages were marked with red dot

31 patterns and 9 sub-lineages reference sequences participated in building the tree of HPV16. The phylogenetic tree showed that all patterns were clustered in lineage A,except NC43 (lineage C). The A branch contained 10 patterns (17 isolates) of sub-lineage A1, 4 patterns (7 isolates) of sub-lineage A3 and 18 patterns (32 isolates) of sub-lineage A4. None of patterns belonged to sub-lineage A2 (Fig. 2).

**Fig. 3:**
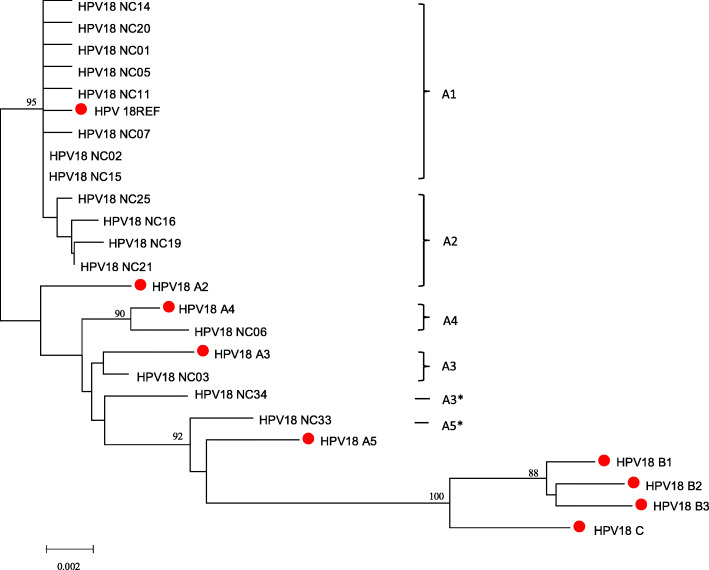
Neighbor-joining phylogenetic tree of HPV-18 patterns based on LCR sequences. Reference sequences of sub-lineages were marked with red dot; *, marked the sub-lineage that the pattern not clearly identified to, but more close to

The tree of HPV18 was composed of 16 patterns and 9 sub-lineages reference sequences. All patterns were identified as Lineage A, of which 8 patterns (24 isolates) were divided into sub-lineage A1, 4 patterns (4 isolates) were classified as sub-lineage A2, 1 pattern was identified as sub-lineage A3 (3 isolates), A4 (1 isolates), respectively. Two patterns (pattern No.34 and No.33) were not clearly identified, while No.33 was more like sub-lineage A5, No.34 was more close to sub-lineage A3 in the tree (Fig. 3).

**Fig. 4:**
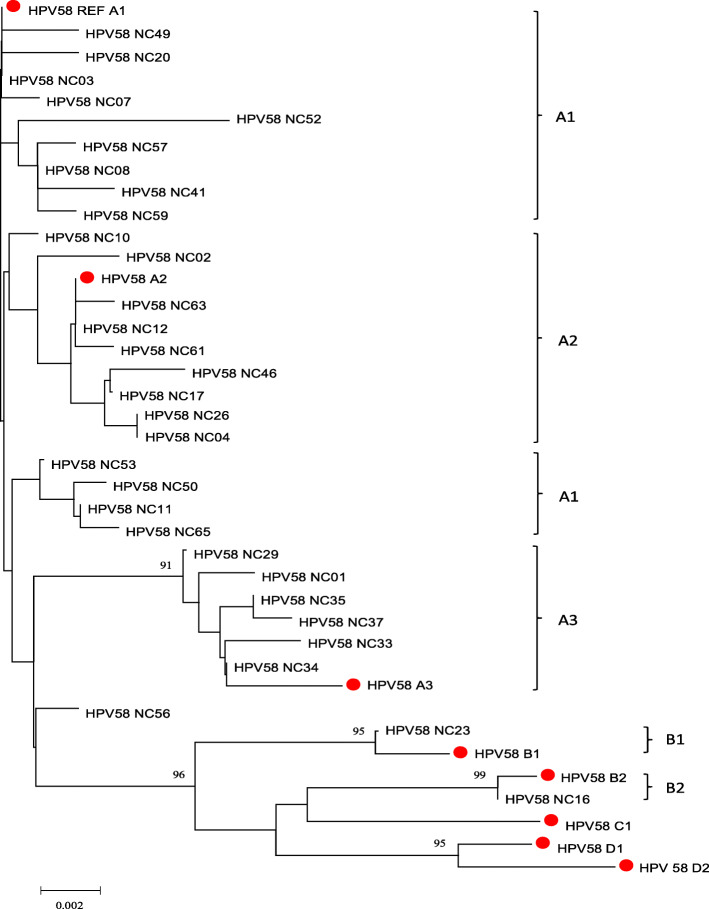
Neighbor-joining phylogenetic tree of HPV-58 patterns based on LCR sequence. Reference sequences of sub-lineages were marked with red dot

39 patterns and 7 sub-lineages reference sequences were selected to build the tree of HPV58. All patterns were gathered in lineage A and B. Lineage A was the most prevalence sub-type of HPV58, including 19 patterns (38 samples) of sub-lineage A1, 10 patterns (16 samples) of sub-lineage A2 and 8 patterns (9 samples) of sub-lineage A3. Three patterns were classified as lineage B, 2 for Sub-B1 and 1 for sub-B2 (Fig. 4).

### Prediction of transcription factor binding sites

JASPAR database was used to investigate the potential binding sites for the transcription factors in the HPV-LCR. Also, it was applied to assess wherther the mutations affected the transcription factor binding sites.

**Fig. 5:**
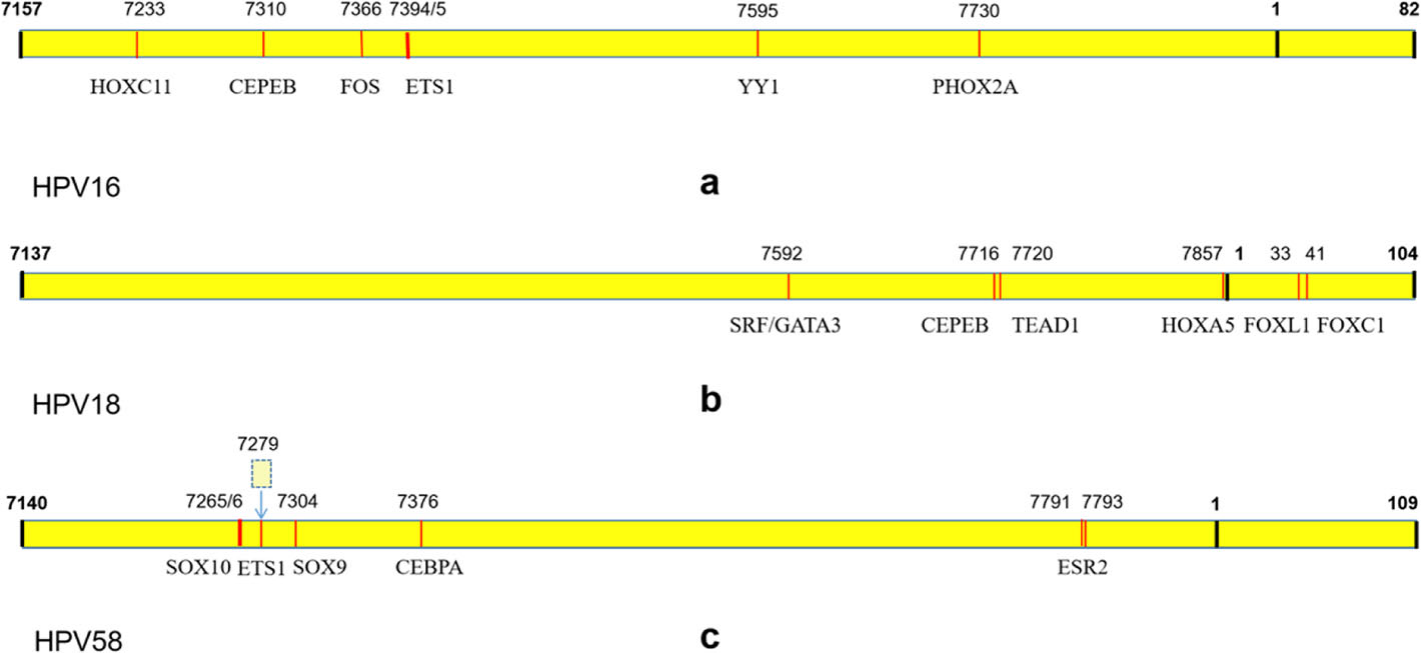
Transcription factors prediction on LCR. (**a**): transcription factors prediction of HPV16. (**b**): transcription factors prediction of HPV18. (**c**): transcription factors prediction of HPV58. The JASPAR database was used to investigate the potential binding sites within HPV-LCR. The yellow strips represent sequence of LCR.: Bold black line marked boundary positions of LCR and initiation sites of genome.: Red line marked variants sites which may effect binding sites of transcription factors.: The block with dash line present the insertion we found in HPV58

For HPV16, the results showed 7 mutations potentially affected the binding sites of transcription factors. A potential binding site of PHOX2A located at nucleotide position 7730 which was detected as the second most variable site of HPV16-LCR. More transcription factors like HOXC11, CEBPB, FOS, ETS1 and YY1 were detected on position 7219, 7310, 7366, 7394/7395 and 7595, respectively (Fig. 5a).

The JASPAR result of HPV18-LCR indicated that 6 variations had potential effects on the TFBS. T7592C was detected in all isolates of HPV18 and might be the binding site of GATA3 and SRF. CEBPB was related to variations C7716T and C7720A. C7716T was also potentially affected the binding site of TEAD1. In addition, nucleotide sites 7857, 33 and 41 potentially affected the binding sites for HOXA5, FOXL1 and FOXC1, respectively (Fig. 5b).

In HPV58-LCR, 5 variations and 1 insertion were found potential effect on TFBS. A sequence of CTTGTCAGTTTC was inserted in the potential binding sites of transcription factor ETS1 between position 7279 and 7280. More TFs like SOX10, SOX9, CEBPA and ESR2 indicated underlying binding change on account of the mutation C7265G, A7304G, A7376G and T7791A/A7793C, respectively (Fig. 5c).

## Discussion

Persistence infection of high-risk HPV shows significant link with cervical intraepithelial neoplasia (CIN) and invasion cervical cancer. HPV variants may have co-diversified with human populations and thus obtain an intrinsic geographical difference on prevalence and infections [26]. The knowledge of epidemicity about HR-HPV is vital for further study of vaccine and cervical cancer therapy in local area.

These three types of HR-HPV showed different prevalence and mutation rates in our study. More HPV16 and HPV58 were detected than HPV18 in this study. Only one HPV16 isolate was completely same with HPV16 REF sequence, while 16 (24.2%) isolates were same with REF sequence of HPV58. It may attributed to the high prevalence and active interaction in viral genome integration and cancer development of HPV16, so the LCR were able to occur and reserve more variations. HPV18 is less popular than HPV16/58 in Southwest China, and we didn’t find as many mutations as those two types in 18. T7592C was a general mutation for HPV18 in this area. It was found in all isolates of HPV18 and also the only variation of 35.3% (12 isolates) HPV18 LCR sequence. HPV58 showed different prevalence through geographical regions. The unusual high prevalence had been reported in East Asia, Africa and some other areas [27]. A few study reported the high prevalence of HPV58 in Japan (8%) and Korea (16%) [28–30]. About the data in China, infection frequency of HPV58 was found 9.4% in Zhejiang [31], 10% in Hong Kong [32] and 10% in Taiwan [33]. In this study, HPV58 occupied 12% of the positive samples. It suggested that further study of vaccine need to consider this type as target.

The distribution of the sub-lineages were basically same with the data had been reported previously. In this study, A lineage accounted for a large proportion of all three types. It has been reported that lineage A related to pathogenicity more than other lineages.

For HPV16, a worldwide phylogenetic analysis indicated that the EUR lineages (A1 ~ A3) were epidemic in many regions. A4 was largely found in Eastern Asia [34]. 56% of HPV16 variants were clustered into sub-lineage A4 which was significantly associated with elevated risk of cervical cancer comparing with A1 ~ 3 [35].

All HPV18 isolated were identified as lineage A. The global data of lineage A showed that sub-lineage A1 predominated in Eastern Asia and Pacific, while sub-lineage A3/A4 were prevalent in many region around the world. The isolates of A5 were detected principally in Africa [36]. Our data showed that HPV18 samples were mostly identified as A1 and a small amount of A2/A3/A4. 70.6% isolates of HPV18 were clustered in sub-lineage A1. Amador-Molina et al. found that the variations of sub-A1 affected Ori function significant higher than the other variants [37]. This effect may be related to the changes found in the keratinocyte enhancer (KE) region of LCR as it has been reported that mutations in this domain affect HPV replication [38].

For HPV58, globally, the sub-lineage A2 was the most widespread variant, whereas sub-lineages A1 and A3 were rarely found out of Asia. A1 were the most prevalence sub-lineage of HPV58 in our study, A2/A3 were also be found. 95.4% of the HPV58 isolates were clustered in lineage A which showed much more relation with CIN2/CIN3+ rather than lineage BCD [30]. We also found 3 variants of lineage B which was rarely observed in East Asia [39].

The HPV LCR which contain the binding sites for both viral and cellular factors, has shown regulatory functions on replication of HPV, transcriptional activity of the E6/E7 and the other interaction through the virus life cycle [10, 13, 28]. Mutations on LCR may influence the binding sites and the function of it. The mutations of some sub-lineages which are more related to pathgenicity, showed potential effect on TFBS in our data. In HPV16, A7730C, variant of sub-lineage A4, showed potentially effect to PHOX2A which is a transcription factor involving in cell proliferation and migration in lung cancer [40]. CEBPB, FOXL1 and HOXA5 were the transcription factors that may be affected in sub-lineage A1 of HPV18. CEBPB is a leucine-zipper transcription factor that regulates growth and differentiation of hematopoietic and epithelial cells. One study based on breast cancer found that CEBPB was a novel transcriptional regulator of CLDN4. The upregulation of CEBPB-CLDN4 signaling caused the migration and invasion of cancer cell [41]. Homeobox A5 (HOXA5) is a member of the homeobox (HOX) family and is upregulated in many types of tumors [42]. Forkhead box L1 (FOXL1) is a member of the Forkhead box (FOX) superfamily and was reported to be dysregulated in various types of cancers. Upregulation of FOXL1 greatly inhibits cell proliferation, migration, and invasion in vitro and tumorigenicity in nude mice [43]. In HPV58, the nucleotide sites 7265 and 7266 were not only the most variable sites in LCR but also the potential binding sites of SOX10 which is a transcription factor of sex determining region Y (SRY)-related high motility group (HMG)-box gene family. SOX10 has been suggested as a useful marker for corresponding tumors [44], although it was usually silenced or downregulated in malignant tumors such as digestive cancers [45] and prostatic carcinoma [46]. The insertion (between 7279 and 7280) which is usually detected in sub-lineage A3, may affect the binding site of ETS1. ETS1 belongs to the large family of the ETS domain family of transcription factors and is involved in cancer progression. Mostly, ETS1 expression is linked to poor survival and contributed to the acquisition of cancer cell invasiveness, EMT (epithelial-to-mesenchymal transition), the development of drug resistance and neo-angiogenesis [47].

## Conclusion

In conclusion, this study investigated the gene polymorphisms, phylogeny, and relevant functional prediction of high-risk HPV-LCR from Southwest China. Although our study showed some limitations on sample capacity and source, it provided more data for understanding the intrinsic geographical relatedness of HPV-16/18/58 variants, the complicated relation among HPV-16/18/58 LCR mutations, transcription factors and carcinogenesis. It also helps performing further study to demonstrate the biological function of HPV-16/18/58 LCR variants and the effect of multiple infection of high-risk HPV on tumor progression. The TFBS we found is still need deeper exploration for the potential of them to be marker in diagnosis and therapy.

## Data Availability

All data generated or analyzed during this study are included in this article and Genbank.

## Abbreviations

DNA: Deoxyribonucleic Acid
HPV: Human Papillomavirus
LCR: Long control region
MEGA: Molecular Evolutionary Genetics Analysis
NCBI: National Center for Biotechnology Information
PCR: Polymerase Chain Reaction
SNP: Single nucleotide polymorphism
TFBS: transcription factor binding site
